# Antioxidative Peptides from Proteolytic Hydrolysates of False Abalone (*Volutharpa ampullacea perryi*): Characterization, Identification, and Molecular Docking

**DOI:** 10.3390/md17020116

**Published:** 2019-02-13

**Authors:** Shudong He, Yi Zhang, Hanju Sun, Ming Du, Jianlei Qiu, Mingming Tang, Xianbao Sun, Beiwei Zhu

**Affiliations:** 1 School of Food Science and Technology, National Engineering Research Center of Seafood, Dalian Polytechnic University, Dalian 116034, Liaoning, China; shudong.he@hfut.edu.cn; 2 School of Food and Biological Engineering, Engineering Research Center of Bio-process of Ministry of Education, Hefei University of Technology, Hefei 230009, Anhui, China; sunhanjv@163.com (H.S.); m15798936158@163.com (J.Q.); tangmingming@mail.hfut.edu.cn (M.T.); sunxianbao@hotmail.com (X.S.); 3 Anhui Province Key Laboratory of Functional Compound Seasoning, Anhui Qiangwang Seasoning Food Co., Ltd., Jieshou 236500, Anhui, China; 4 Department of Food Science and Agricultural Chemistry, McGill University, Ste-Anne-de-Bellevue, QC H9X 3V9, Canada; yi.zhang10@mail.mcgill.ca

**Keywords:** false abalone, antioxidative peptides, ABTS^+•^ scavenging activity, MPO, docking

## Abstract

Antioxidative peptides were produced from false abalone (*Volutharpa ampullacea perryi*) using enzymatic hydrolysis. Trypsin produced the most bioactive hydrolysates with the highest scavenging ABTS^+•^ free radicals compared to pepsin, alcalase, neutrase, and flavourzyme. The response surface methodology studies on trypsin hydrolysis indicated that the hydrolysis temperature, time, and pH were interacted with each other (*p* < 0.05), and the optimal conditions were hydrolysis at 51.8 °C for 4.1 h, pH 7.7 and the maximum predicted hydrolysis degree was 13.18% and ABTS^+•^ scavenging activity of 79.42%. The optimized hydrolysate was subjected to ultrafiltration fractionation, and the fraction with MW < 3 kDa showed the highest ABTS^+•^ scavenging activity. There were 193 peptide sequences identified from this peptide fraction and 133 of them were successfully docked onto human myeloperoxidase (MPO), an enzyme involved in forming reactive oxidants *in vivo*. The highest scored peptide, no. 39, consists of DTETGVPT. Its structure and molecular interactions with MPO active site were compared with previously characterized peptide hLF1-11. The interactions between peptide no. 39 and MPO include electrostatic charge, hydrogen bonds, and covalent bonds. The antioxidative peptide produced in this research may exert antioxidant activity in vivo due to its potential inhibition effect on MPO.

## 1. Introduction

Compared with pacific abalone (*Haliotis discus*), which is a highly valued marine shellfish, the false abalone (*Volutharpa ampullacea perryi*), another deep-sea snail gastropod, is much cheaper but of similar appearance and nutrition value to that of *Haliotis discus* [1]. False abalone is abundantly found in the North Pacific, especially some coasts of China and Japan, where it is consumed as food [2]. Its edible parts contain approximately 50% protein (dry weight) [3], which makes it a potential resource for developing bioactive peptides to increase its commercial value. Marine-derived peptides have been well studied to be used as bioactive components in functional foods or nutraceuticals and pharmaceuticals. However, bioactive peptides produced from false abalone are rarely reported.

Enzymatic hydrolysis for producing peptides basically includes hydrolysis at optimal conditions, termination of hydrolysis, filtration, fractionation or purification, and freeze-drying [4]. Proteolytic enzymes, either endogenous or exogenous, are a group of enzymes that cleave peptide bonds in protein matrices to generate peptide mixtures with different sizes, amino acid compositions and sequences, as well as peptide structures [5]. However, since each proteolytic enzyme has a different degree of specificity, the selection of proteolytic enzymes certainly determines the bioactivity of the produced peptides.

Antioxidative peptides have broad health benefits by controlling oxidative stress, which plays a role as a promoter for some chronic diseases, such as diabetes, atherosclerosis, arthritis, and cancer [6]. The antioxidant effect of peptides can result from the scavenging of reactive oxygen species (ROS) and chelating transition metal ions [7]. Although the exact mechanism of the antioxidant activity of peptides has not been clearly understood, various research studies have found that certain composed amino acids and their locations, as well as the configuration of the peptide, are involved in the interaction with radicals. Such interactions can be evaluated using in silico molecular docking of peptides with myeloperoxidase (MPO). MPO is abundantly expressed in neutrophils and participates in innate immune defense by forming reactive oxidants contributing to tissue damage; therefore, MPO is used in drug design and development for high-throughput screening of compounds based on the enzyme inhibition mechanism [8]. For example, Guerra-Vargas et al. used MPO (Protein Data Bank (PDB) ID: 1DNU) as a target to further study the synthesized new phenolic compounds that serve as antioxidants [9]. Van der Does et al. used human MPO (PDB ID: 3F9P) to study the inhibition effect of a novel antimicrobial peptide on MPO activity [10].

The objectives of the present research are: (i) to optimize the production of antioxidative peptides from false abalone using proteolytic hydrolysis; (ii) to characterize their antioxidant activity of peptide fractions by spectroscopic evaluations of the scavenging of ABTS^+•^ (the diammonium salt of 2,2′-azino-bis(3-ethylbenzothiazoline-6-sulfonic acid)); (iii) to identify the amino acid sequences of the optimized peptides using LC-MS/MS; and (iv) to understand the mechanism via the molecular docking model using MPO.

## 2. Results and Discussion

### 2.1. The Selection of Proteolytic Enzymes

**Figure 1:**
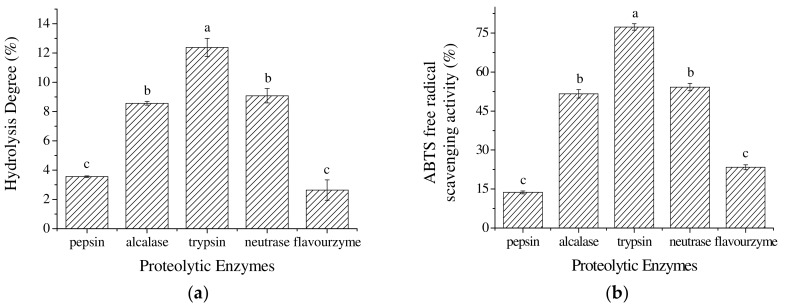
The effect of proteolytic enzymes on the (**a**) hydrolysis degree and (**b**) ABTS^+•^ free radical scavenging activity of hydrolysates (the amounts of proteins: 1.0 mg/mL). Different letters in one panel mean significant difference (*p* < 0.01).

Proteolytic enzymes break down peptide bonds to produce protein hydrolysates, which consist of peptides and amino acids. The commercially used proteolytic enzymes are usually protease cocktails, such as the alcalase, neutrase, and flavourzyme that have low specificity on the amino acids residues; besides, trypsin (EC 3.4.21.4), and pepsin (EC 3.4.23.1) are also widely used and they are highly specific to basic and aromatic amino acids residues, respectively [11]. As mentioned previously, the specificity of enzymes determines the size and the sequence of the peptides, and their antioxidant activity. Figure 1a shows the effect of five proteolytic enzymes on the hydrolysis degree of false abalone hydrolysates. Trypsin produced hydrolysates with the highest hydrolysis degree (12.38 ± 0.62%) (*p* < 0.01), followed by non-specific neutrase (9.08 ± 0.50%), and alcalase (8.57 ± 0.13%). Hydrolysates produced from flavourzyme, which is usually used to produce hydrolysates as flavor compounds, had the lowest hydrolysis degree (2.63 ± 0.70%), which had no significant difference with the pepsin-produced hydrolysates (hydrolysis degree of 3.55 ± 0.05%) (*p* > 0.01). Hou et al. reported that trypsin produced Alaska pollock protein hydrolysates with the highest hydrolysis degree compared with nine other proteases due to the substrate specificity on lysine and arginine residues of the trypsin, and that trypsin is usually served in higher activity and purity than other proteases as one kind of serine protease [12]. The effect of the five proteolytic enzymes on the ABTS^+•^ free radical scavenging activity is shown in Figure 1b. Trypsin-produced hydrolysates had the highest ABTS^+•^ scavenging activity of 77.32 ± 1.25% (*p* < 0.01), with the amount of proteins of 1.0 mg/mL, and its IC50 value calculated to be 0.69 mg/mL. Similarly, Mao et al. found that casein hydrolysates prepared with alcalase and trypsin had significantly higher DPPH (2,2-diphenyl-1-picrylhydrazyl) free radical scavenging capacity than those prepared with pepsin and flavourzyme [13].

The hydrolysis degree and ABTS^+•^ scavenging activity of produced hydrolysates followed similar trends, which suggest that the increase of hydrolysis degree may increase the antioxidant properties of hydrolysates to some extent. Higher hydrolysis could result in a larger exposure of the active amino acids residues that are capable of reacting with oxidants [14]. However, extensive hydrolysis could have a negative impact on the functional properties of hydrolysates [15]. Based on the results shown in Figure 1, trypsin was selected to produce oxidative hydrolysates for subsequent optimization studies.

### 2.2. Optimum Hydrolysis Conditions

The hydrolysis degree (Response 1) and ABTS^+•^ free radical scavenging activity (Response 2) are two important indexes of antioxidative hydrolysates produced from enzymes. These two responses obtained under various conditions designed by face-centered central composite design (F-CCCD) are shown in Table 1. It indicated that hydrolysis conditions such as temperature, time, and pH could influence the hydrolysis and the bioactive property of hydrolysates produced with trypsin. 

Response surface analysis data for the hydrolysis degree of hydrolysates (Table 2; Response 1 section) indicated a significant model (*p* < 0.0001), as well as significant (*p* < 0.05) linear effects A, B, C; interaction term AB; and quadratic effects A^2^, B^2^, C^2^ for the fitted model, as seen in Equation (1):

Hydrolysis degree (%) = 12.44 + 0.52A + 0.39B + 2.68C − 3.39A^2^ − 3.65B^2^ − 2.07C^2^ − 0.78AB + 0.12AC − 0.01BC. (1)

**Figure 2:**
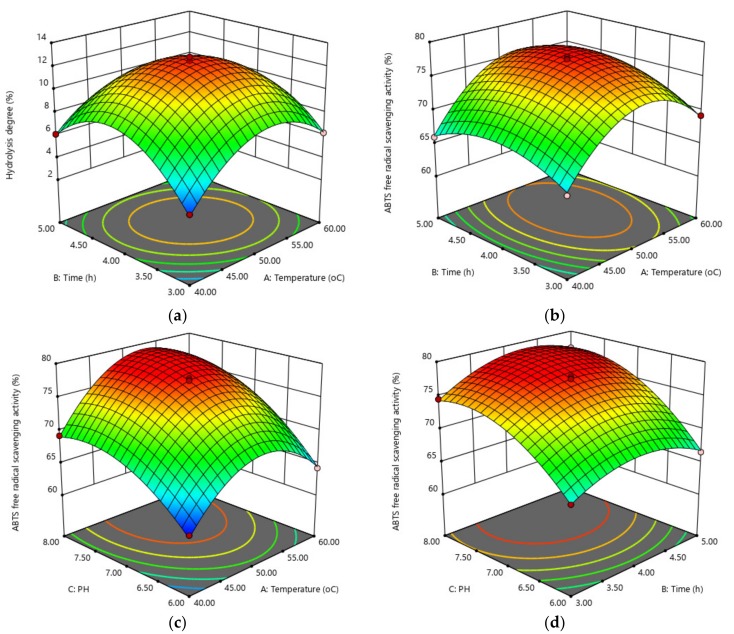
Three-dimensional (3D) response surface plots. (**a**) Hydrolysis degree (%) as a function of temperature (°C) × time (h); (**b**) ABTS^+•^ scavenging activity (%) as a function of temperature (°C) × time (h); (**c**) ABTS^+•^ scavenging activity (%) as a function of temperature (°C) × pH; (**d**) ABTS^+•^ scavenging activity (%) as a function of time (h) × pH.

The lack of fit was not significant (*p* = 0.1000), indicating that the model fitted the data very well. The maximum predicted hydrolysis degree of 13.34% was obtained according to Equation (1) under the following conditions: temperature 50.82 °C, time 4.04 h, and pH 7.65. As shown in Figure 2a, hydrolysis temperature (A) and time (B) interacted strongly (*p* = 0.0027) with respect to the hydrolysis degree. The highest hydrolysis degree was observed at hydrolysis temperature of 50 °C for 4 h. Higher temperature combined with longer hydrolysis time negatively influenced the hydrolysis degree. The reason could be the inactivation effect on trypsin with temperatures above 50 °C, as long treatment time at high temperatures would reduce the availability of the enzyme, increase the concentration of produced peptides that may inhibit the hydrolysis reaction, and decrease the concentration of peptide bonds that limit the reaction speed. Similar observations have been reported for the yellowfin tuna hydrolysates produced using alcalase [16] and β-lactoglobulin hydrolysates produced using trypsin [17]. 

ABTS^+•^ scavenging activity is of prime importance for antioxidative hydrolysates because it is used as a parameter to evaluate the antioxidant properties. As shown in Table 2 (Response 2 section), the corresponding *p* < 0.0001 indicated the model fitted well with the experimental data, as expressed in Equation (2):

ABTS^+•^ scavenging activity (%) = 77.49 + 2.32A + 0.88B + 4.63C − 6.39A^2^ − 2.91B^2^ − 3.38C^2^ + 0.57AB + 0.97AC − 0.66BC. (2)

The *p* values for all the effects, i.e., linear effects A, B; interaction terms AB, AC, BC; as well as quadratic effects A^2^, B^2^, C^2^ were significant (*p* < 0.05). The lack of fit was not significant (*p* = 0.0673), which confirmed an excellent fitting. The optimum predicted ABTS^+•^ scavenging activity of 79.62% was obtained, according to Equation (2), under the following conditions: temperature 52.5 °C, time 4.26 h, and pH 7.75. Figure 2b shows the significant interaction between hydrolysis temperature (A) and time (B) (*p* = 0.0270). At lower temperatures, the ABTS^+•^ scavenging activity increased very slowly when hydrolysis time increased. At higher temperatures (>50 °C), as shown in the red surface on the top of the three-dimensional (3D) diagram, the ABTS^+•^ scavenging activity of hydrolysates increased significantly with the increase of hydrolysis time from 3.5 to 4.5 h. As seen in Figure 2c, the ABTS^+•^ scavenging activity increased when hydrolysis pH increased. A higher pH combined with moderate hydrolysis temperatures favored the production of hydrolysates with high ABTS^+•^ scavenging activity. Figure 2d shows that at higher pH values (pH 7–8), the ABTS^+•^ scavenging activity increased significantly with the increase of hydrolysis time. The hydrolysis pH had more influence than hydrolysis temperature and time. This might be due to the trypsin used being sensitive to the pH and active at basic pH conditions. A previous study found that hydrolysis time, temperature, and enzyme/substrate ratio had interaction effects on the ABTS^+•^ scavenging activity of fish gelatin hydrolysates prepared using papain [18]. 

Then, with respect to both hydrolysis degree and antioxidative activity, the first partial derivatives of the regression models were applied to optimize the enzymatic hydrolysis by combining the Equations (1) and (2). Hence, the optimum conditions of hydrolysis temperature at 51.81 °C for 4.13 h at pH 7.7 were determined to produce antioxidative hydrolysates using trypsin, and the maximum predicted hydrolysis degree was 13.27% with ABTS^+•^ scavenging activity of 79.54%. To verify the model, the practical conditions of hydrolysis temperature at 51.8 °C for 4.1 h at pH 7.7 were applied because of the control accuracy of the equipment. New hydrolysates produced under these conditions showed a hydrolysis degree of 13.18 ± 0.31% and ABTS^+•^ scavenging activity of 79.42 ± 0.33%, which showed no significant difference from the predicted data (*p* > 0.05). The optimized hydrolysates were subjected to fractionation using ultrafiltration to determine the effect of fraction MW on their anti-oxidant properties.

### 2.3. The Effect of MW

**Figure 3:**
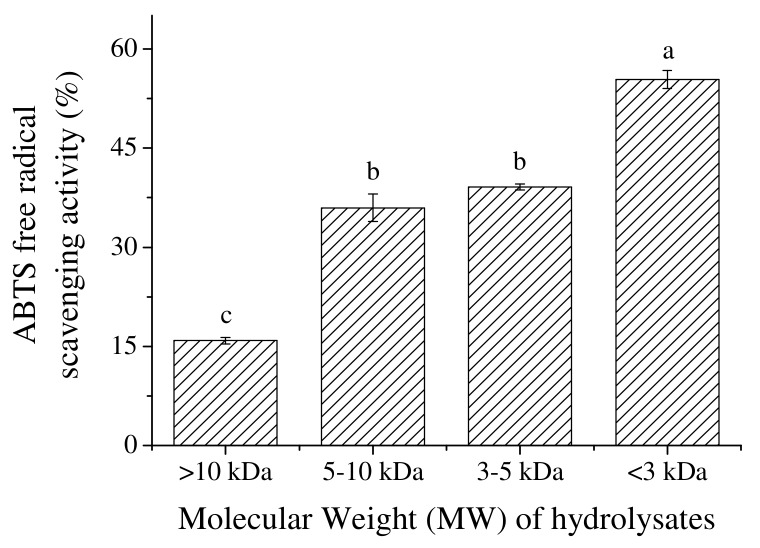
The effect of fractions with different MW from optimized hydrolysates on the ABTS^+•^ scavenging activity.

The molecular weight of protein hydrolysates that resulted from the enzymatic hydrolysis degree was highly related with their bioactivity. After the ultrafiltration, all the protein hydrolysates were diluted. Then the protein concentration of any fraction was adjusted to 0.1 mg/mL prior to the determination of antioxidant properties. As seen in Figure 3, the ABTS^+•^ scavenging activity of hydrolysate fractions increased significantly from MW of <10 kDa to MW of 5–10 kDa (*p* < 0.01), and the increase of activity between hydrolysate fraction 3–5 kDa and <3 kDa was gradual. The hydrolysate fraction with a MW < 3 kDa was found to have the highest ABTS^+•^ scavenging activity of 55.37 ± 1.33% (*p* < 0.01) at a low protein concentration of 0.1 mg/mL. Since the IC50 value of the <3 kDa hydrolysate fraction was calculated to be 0.09 mg/mL, which was about 7.7 times higher than that of the initial tryptic hydrolysate, the results indicated that the lower the MW of the hydrolysates, the higher the ABTS^+•^ scavenging activity. Protein hydrolysates from fish, beans, milk, etc., with a MW range of 0–3 kDa, have been reported with higher antioxidant properties [19,20,21]. Besides, peptides with lower MW are easier to cross the intestinal barrier and exert the antioxidant effect and can interact more effectively with free radicals [22], which favor their usage as pharmaceuticals. 

### 2.4. Identification of Peptide Sequences by LC–MS/MS

The fraction with MW of < 3 kDa was loaded onto LC–MS/MS to characterize their sequences of the composed amino acids. There were 193 peptides recognized in the hydrolysate fraction, as listed in Supplementary Table S1. Results showed that all peptide sequences were made of 6–26 amino acids residues. Previous research has found that bioactive peptides usually contain 2–20 amino acid residues [23,24]. In the current study, the molecular mass of the peptide sequences ranged from 699 to 2560 Da, which also confirmed the effective fractionation using ultrafiltration to produce peptides with MW < 3 kDa. 

The peptide sequences in the MW < 3 kDa had various amino acid residues at their N- or C-terminals. Trypsin could specifically cleave the C-terminal to arginine (R) or lysine (K) residues. There were 81 peptide sequences (42% of the total peptide sequences) with R or K residues on the C-terminal, which implied the efficiency of enzymatic hydrolysis using trypsin. It has been reported that trypsin-generated peptides from royal jelly protein hydrolysates had high antioxidant and antimicrobial activities [25,26]. The presence of peptide sequences with other residues on the C-terminal may have been caused by the impurity of trypsin enzymes, or autohydrolysis may have occurred with the endogenous digestive enzymes from false abalone, or the breakdown of random peptide bonds at heating treatment (i.e., 51.8 °C for 4.1 h), or the matching degree of the MS/MS database. The diversity of the remaining residues on the peptide sequence terminals contributed to the antioxidant properties. For instance, proline (P) residues at both terminals were found in peptides that had both antioxidant and α-amylase inhibitory activities [24]; the presence of the N-terminal histidine (H) was shown to exert antioxidant protection [27].

Previous research studies have found that antioxidative peptides usually contain hydrophobic amino acids, such as glycine (G), alanine (A), valine (V), leucine (L), isoleucine (I), proline (P), phenylalanine (F), methionine (M), and tryptophan (W) [28]. Those residues were found in almost all the 193 characterized peptide sequences, suggesting that they could enhance the presence of peptides in the water–lipid interaction phase to facilitate the scavenging of free radicals according to the previous study [29]. Among these hydrophobic amino acids, leucine (L) was found in the majority of the characterized peptides. This was in agreement with research on peptides from buffalo and bovine casein hydrolysates with high antioxidant properties [30]. It might be due to the long aliphatic side chain from leucine residues that contributed to the interaction with the acyl chains of fatty acids [31]. Besides, amino acid residues, such as histidine (H), and cysteine (C) are also found in the peptides sequences, which have been reported with relation to radical scavenging activity due to their special characteristics, such as the imidazole group in histidine and the sulfur hydrogen donated by cysteine [32].

### 2.5. Molecular Mechanism

There were 133 among 193 identified peptide sequences successfully being docked with the MPO enzyme, which suggested that 69% of peptides in the false abalone hydrolysate with MW < 3 kDa had potential antioxidant activity in vivo. These 133 peptide sequences had a total of 820 docking poses (data not shown), and based on the “score ligand poses” function with calculations from LigScore1, LigScore2, PLP1, PLP2, Jain, PMF, PMF04, and CDOCKER scores algorithms, 107 best poses from 107 peptides were returned and listed in Table 3, which were ordered by the consensus scores based on the scores that had been previously computed. Peptide no. 39 with 8 amino acid residues Asp–Thr–Glu–Thr–Gly–Val–Pro–Thr (DTETGVPT) had the best consensus score. There were another 39 peptide sequences in number had consensus scores of 2, and the amounts of peptides with consensus scores of 1 and 0 were 53 and 14, respectively. The reference bioactive peptide hLF1-11, made up of 11 amino acid residues (Gly–Arg–Arg–Arg–Arg–Ser–Val–Gln–Trp–Cys–Ala, GRRRRSVQWCA), has been characterized in structure [10,33]. The docking study of this hLF1-11 with MPO showed that several electrostatic interactions were involved so that hLF1-11 could occupy the active site of MPO and block its catalytic activity [10]. In this study, we used hLF1-11 to compare with the docked peptides, especially peptide no. 39, using the docking procedure described in Section 3.9, to help to illustrate the molecular mechanism of peptide no. 39 for its antioxidant effect.

Reference peptide hLF1-11 and peptide no. 39 had similar interaction energy around a value of −120. As shown in Table 3, the interaction sites between peptide no. 39 and amino acids residues of MPO were similar to those between the reference peptide and MPO. For the LF1-11, the interactions with MPO were amino acids ARG 31 on the A chain, VAL30, ARG31, and TRP32 on the B chain, ARG323 and LYS505 on the C chain, and ALA152, CYS153, ILE160, and LYS505 on the D chain. Nevertheless, peptide no. 39 seemed to have a stronger binding with MPO than LF1-11, as not only the mentioned amino acids above, but also the TRP32 on the A chain of MPO, ALA28, PHE29 on the B chain, ILE160 on the C chain, and THR159 and ARG323 on the D chain were also involved in the peptide no. 39 and MPO interactions. The binding of peptide no. 39 onto these amino acid residues may have interrupted the rigid hydrogen bond, electrostatic interaction, and covalent bond network in the active site that are important for catalysis by MPO [34,35].

**Figure 4:**
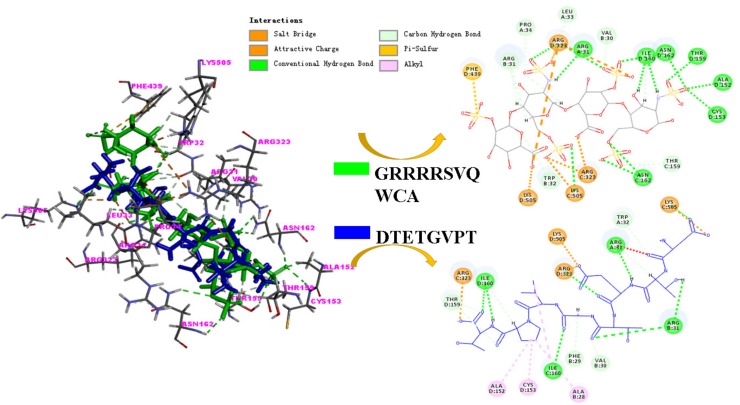
Molecular docking model of the interactions between hLF1-11(GRRRRSVQWCA) and MPO, as well as between peptide no. 39 (DTETGVPT) with the active sites of MPO (Protein Data Bank (PDB) ID: 3F9P). The structure of hLF1-11 and peptide no. 39 are shown in green and blue stick representations, respectively, in the docking model shown on the left. The amino acid residues around the MPO active site that are involved in the interactions are labelled in magenta color. The interactions between peptides (hLF1-11 or peptide no. 39) and MPO active site are labelled in different colors for better visualization (charge interactions in orange color, conventional hydrogen bond in emerald green color, carbon hydrogen bond in light green color, pi sulfur interaction in light orange color, and alkyl interaction in light pink color).

As shown in Figure 4, there is structure similarity observed between reference hLF1-11 and peptide no. 39. Peptide no. 39 physically suited well in the docking pocket of MPO with the similar length. It could be observed from the docking model in Figure 4 that peptide no. 39 was able to block the active sites of MPO to inhibit the catalytic activity of MPO. From the structure comparison of hLF1-11 and peptide no. 39, the interactions involved were similar. The charge interactions were found between peptides (hLF1-11 and peptide no. 39) and MPO residues ARG323 and LYS505, for example, via the –COO group of peptide no. 39 and –NH group of arginine residue in MPO. Peptide no. 39 also formed hydrogen bonds from the −C=O and –NH groups of peptides to the ARG31 and ILE160 residues in MPO, and these two residues were also involved in hydrogen bond formation between reference hLF1-11 and MPO pocket. C–H bonds were predicted to be formed between peptide no. 39 and amino acid residues VAL30, TRP32, and THR159, which were also associated in the C–H bond interactions between reference and MPO. The difference in the predicted interactions was that 1 pi sulfur interaction was predicted between reference hLF1-11 and PHE439 in MPO, as well as 3 acyl interactions predicted between peptide no. 39 and ALA152, CYS153, and ALA28 in MPO. The molecular docking (Figure 4) indicated that peptide no. 39 (DTETGVPT) was similar in structure as well as the interactions with amino acid residues in the MPO active site, which made it possible for peptide no. 39 to perform antioxidative activity in vivo.

## 3. Materials and Methods 

### 3.1. Materials and Reagents

Fresh false abalone were purchased from the local market in Dalian, China. The water content and protein content of the false abalone samples were 77.53 ± 2.13% and 18.24 ± 1.03% using methods GB/T5009.3-2016 and GB/T5009.5-2016, respectively. Five proteases including pepsin (≥400 units/mg protein), alcalase (≥200 units/mg protein), trypsin (≥2500 USP units/mg protein), neutrase (≥600 units/mg protein), and flavourzyme (≥300 units/mg protein) were purchased from Sinopharm Chemical Reagent Co., Ltd., Shanghai, China. Other chemicals and reagents were of analytical degree.

### 3.2. Proteolytic Hydrolysis of False Abalone

False abalone samples were blended in different buffer solutions (pH 2.0, 7.0, 8.0, and 10.0) at the ratio of 3% (w/v). Five proteases were added at a concentration of 7% (w/v) individually. The commercial recommended hydrolysis temperatures and pH values for each protease were applied as: pepsin at 37 °C, pH 2.0; alcalase at 50 °C, pH 10.0; trypsin at 50 °C, pH 8.0; neutrase at 50 °C, pH 7.0; and flavourzyme at 55 °C, pH 7.0. After 3 h hydrolysis, the mixtures were treated at 100 °C for 10 min to inactivate the proteases, followed by centrifugation at 8000 rpm for 15 min. The supernatants were collected as false abalone hydrolysates. 

### 3.3. Determination of Protein Concentration and Hydrolysis Degree

The protein concentration of hydrolysate was measured using the BCA Protein Assay Reagent Kit (Beyotime Biotechnology Co., Shanghai, China) following the instructions. The hydrolysis degree of hydrolysate was determined based on the content of amino nitrogen using the formol-titration method according to GB/T5009.39-2003. Briefly, 1 mL hydrolysate sample was diluted with 60 mL distilled water and was then titrated to pH 8.2 using 0.05 M NaOH solution (V_a_). Formaldehyde solution of 10 mL was added, and the mixture was titrated continuously to pH 9.2 using a 0.05 M NaOH solution (V_b_). The NaOH solution consumed after formaldehyde addition was V_sample_ (i.e., V_b_ – V_a_). Blank was performed using distilled water instead of hydrolysates, and the NaOH solution consumed after formaldehyde addition was V_blank_. The hydrolysis degree was calculated using Equations (3) and (4) with samples before and after hydrolysis. Total amino nitrogen was measured using samples after complete hydrolysis with acid treatment for 12 h.

X (Amino nitrogen content, g/100 mL) = (V_sample_ − V_blank_) × c × 0.014 × 100 (3)

Hydrolysis degree (%) = (X_before hydrolysis_ – X_after hydrolysis_)/X_complete hydrolysis_ (4)

where c is the concentration of NaOH solution used, and 0.014 is the nitrogen content equivalent to 1 mL 1 M NaOH solution.

### 3.4. Antioxidant Activity Determination of Hydrolysates Using ABTS^+•^ Assay

The ABTS^+•^ free radical scavenging activity was determined using the method as described by Li et al. [36] with slight modification. Briefly, ABTS^+•^ free radical was generated in darkness using 10 mL of 7 mM ABTS and 176 µL of 130 mM potassium persulfate for 16 h. The ABTS^+•^ solution was diluted with 60% ethanol to an absorbance of 0.70 ± 0.02 at 733 nm. A hydrolysate sample of 1 mL was mixed with 3.9 mL ABTS^+•^ solution, and the absorbance was measured after 10 min at 733 nm (A_sample_). Blank was measured using ethanol instead of the hydrolysate sample, and the absorbance measured as A_blank_. ABTS^+•^ free radical scavenging activity was calculated using Equation (5):

ABTS^+•^ free radical scavenging activity (%) = (A_sample_ − A_blank_)/A_sample_ × 100%. (5)

### 3.5. Selection of Proteolytic Enzyme

The false abalone hydrolysates produced using five different proteolytic enzymes were subjected to 3.3 and 3.4 to select the best enzyme that produced the highest hydrolysis degree and ABTS^+•^ free radical scavenging activity, which was further used in 3.6 for the optimization of the optimal hydrolysis conditions.

### 3.6. Response Surface Methodology

A three-factor face-centered central composite design (F-CCCD) was applied with hydrolysis temperature (°C), hydrolysis time (h), and hydrolysis pH as independent variables, and hydrolysis degree (%) and ABTS^+•^ free radical scavenging activity (%) as responses. The three uncoded levels corresponding to the codes (−1, 0, +1) of the three independent variables were chosen based on preliminary testing. Using the 17 experimental combinations, the following second-degree polynomial model was fitted (Equation 6):

Y = β_0_ + β_1_A + β_2_B + β_3_C + β_11_A^2^ + β_22_B^2^ + β_33_C^2^ + β_12_AB + β_13_AC + β_23_BC (6)

where Y is the estimated response; A, B, C are independent variables, i.e., hydrolysis temperature, hydrolysis time, and hydrolysis pH, respectively; β_0_ is the intercept coefficient; β_1_, β_2_, and β_3_ are the linear coefficients; β_11_, β_22_, and β_33_ are the quadratic coefficients; and β_12_, β_13_, β_23_ are the linear by linear interaction coefficients. The model includes linear, quadratic, and interaction coefficients. 

### 3.7. Fractionation of the Optimized Hydrolysates

The optimized hydrolysates produced at the optimal conditions were fractionated using a polyethersulphone (PES) ultrafiltration membrane (Millipore Co., USA) with a MWCO of 10, 5, and 3 kDa. Four fractions were obtained with a MW of < 3, 3–5, 5–10, and >10 kDa. These fractions were analyzed for ABTS^+•^ free radical scavenging activity, and the fraction with the highest activity was subjected to 3.8.

### 3.8. LC–MS/MS Assay

The peptide sequences in the hydrolysate fraction were identified using LC–MS/MS. The LC system was equipped with an RP-C18 column (125 mm × 3 mm, particle size 5 μm, Guangzhou FLM Scientific Instrument Co., Ltd., Guangzhou, China), and was operated with mobile phases: solvent A (0.1% (v/v) formic acid in water) and solvent B (acetonitrile supplemented with 0.1% (v/v) formic acid). The column was equilibrated with 95% solvent A. Samples were loaded from the autosampler into the column and then separated with gradient conditions (B%): 0–30 min 4–50%, 30–34 min 50–100%, and 34–35 min 100%. The MS/MS analysis was performed in quadruple using an Orbitrap mass spectrometer (Q Exactive mass spectrometer, Thermo Fisher Scientific Co., Bremen, Germany). Full scan spectra were acquired over m/z 350–2000 and 10 MS2 scans were selected. The software packages of Mascot 2.2 and Uniprot database were used to process and analyze the data.

### 3.9. Molecular Docking

The molecular docking studies were based on the crystal structure of the human MPO (PDB ID: 3F9P) at a resolution of 2.93 Å [37]. The molecular mechanism of peptide interaction with MPO was performed using advanced molecular docking software, Discovery Studio 2017R2 (Accelrys, San Diego, CA, USA) by the CDOCKER model. During the algorithm, the receptor is set as rigid while the peptides (ligands) can be flexible. The “Receptor–Ligand Interactions” performance parameters were set as: (i) Top hits—10, Pose cluster radius—0.5; (ii) Random conformations—10, Dynamics steps—1000, Dynamics target temperature—1000, Include electrostatic interactions—True; (iii) Orientations to refine—10, Maximum bad orientations—800, Orientation vdW energy threshold—300; (iv) Simulated annealing—True, Heating steps—2000, Heating target temperature—700, Cooling steps—5000, Cooling target temperature—300; (v) Forcefield—CHARMm (Chemistry at HARvard Macromolecular Mechanics), Use full potential—False, Ligand partial charge method—Momany–Rone, Final minimization—full potential, Final minimization gradient tolerance—0, Final minimization gradient tolerance—0, Prepare input receptor—True, Grid extension—8.0, Random number seed—314159; (vi) Parallel processing—True, Batch size—1. 

**Figure 5:**
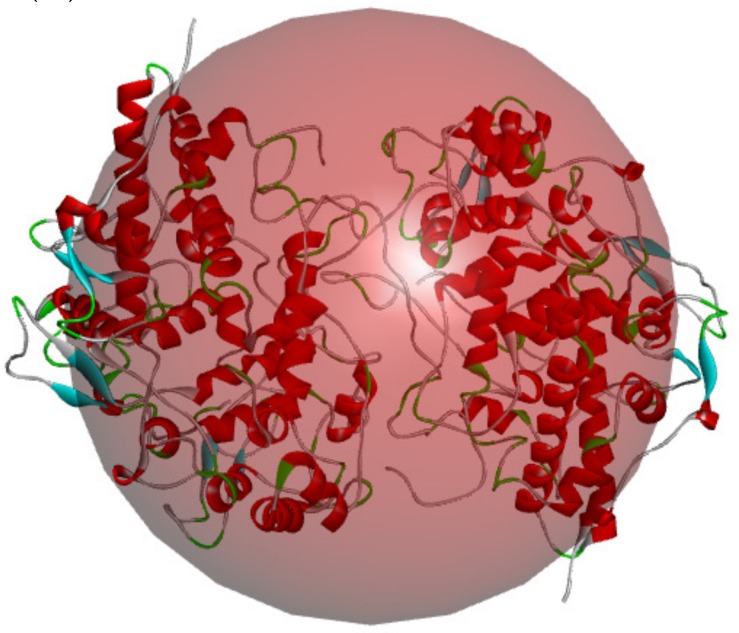
The docking pocket of human MPO (PDB ID: 3F9P).

Before docking, the MPO structure was treated with the Prepare Protein program in Discovery Studio to build loops, minimize the energy, and protonize and remove water molecules. As shown in Figure 5, the docking pocket was defined based on the active sites of MPO described previously, and the human lactoferrin-derived peptide hLF1-11 was used as the reference peptide [10]. Prior to the docking, the identified 193 peptides were minimized in energy using the following parameters: (i) Algorithm—Smart minimizer, Max steps—200, RMS gradient—0.1, Energy change—0, Save results frequency—0; (ii) Nonbond list radius—14, Nonbond higher cutoff distance—12, Nonbond lower cutoff distance—10; (iii) Electrostatics—Automatic. 

### 3.10. Statistical Analysis

Design Expert version 8.0 (Stat-Ease Inc., Minneapolis, MN, USA) was used to perform surface response analysis, including the ANOVA decomposition and significance tests for model coefficients, the optimization of the response as a function of the independent variables, and the plotting of the fitted surface. Triplicates were performed, and the results were given as mean ± SD. The significance was analyzed using SPSS 11.5 (SPSS Inc, Chicago, IL, USA) with a significance level of *p* < 0.01.

## 4. Conclusions

The false abalone hydrolysates produced from proteolytic enzymes have antioxidant properties. Trypsin was found to produce hydrolysates with the highest hydrolysis degree and the highest ABTS^+•^ free radical scavenging activity than other proteases such as pepsin, alcalase, neutrase, and flavourzyme. The optimization of the use of trypsin to produce hydrolysates was of practical importance. Hydrolysate fractions with lower MW showed higher ABTS^+•^ free radical scavenging activity, and 193 peptide sequences were characterized using the LC-MS/MS. There were 133 peptides successfully docked onto the MPO enzyme and 107 of them were characterized with interactions. Peptide no. 39, with amino acid sequence of DTETGVPT, showed the highest consensus score, and its structure as well as the molecular interactions with the amino acid residues in active sites of MPO were similar to a previously characterized peptide hLF1-11. The predicted interactions between peptide no. 39 and MPO included charge interactions, hydrogen bonds, and covalent bonds, which suggested peptide no. 39 might be a potential inhibitor for MPO. Thus, our findings suggested the peptides in the produced antioxidative hydrolysate may exert antioxidant activity *in vivo*, which have application potential in the development of pharmaceuticals and functional foods.

## Figures and Tables

**Table 1:** The experiment data for the hydrolysis degree and ABTS^+•^ scavenging activity of false abalone hydrolysates produced by trypsin using face-centered central composite design (F-CCCD).

Run	Independent Variables			Responses	
	A: Temperature (^o^C)	B: Time (h)	C: pH	Response 1: Hydrolysis Degree (%)	Response 2: ABTS^+•^ Scavenging Activity (%)
1	50	4	7	12.44	77.56
2	50	4	7	12.81	77.49
3	40	5	7	6.11	65.99
4	50	5	8	10.09	77.34
5	50	3	8	9.22	74.53
6	50	4	7	12.23	77.28
7	60	3	7	6.24	69.25
8	50	3	6	3.33	66.37
9	40	4	6	4.03	62.15
10	60	5	7	5.37	72.39
11	60	4	8	10.16	75.23
12	50	5	6	4.25	66.57
13	50	4	7	12.35	77.85
14	60	4	6	5.06	64.25
15	50	4	7	12.35	77.26
16	40	3	7	3.88	65.14
17	40	4	8	8.67	69.25

**Table 2:** Statistical summary of the surface response analysis.

Source	Response 1			Response 2		
	Mean Square	*F* Value	*p* Value	Mean Square	*F* Value	*p* Value
Model	22.13	189.74	<0.0001	56.43	334.76	<0.0001
A	2.14	18.37	0.0036	43.20	256.27	<0.0001
B	1.24	10.63	0.0138	6.13	36.34	0.0005
C	57.62	493.99	<0.0001	171.22	1015.74	<0.0001
AB	2.40	20.60	0.0027	1.31	7.78	0.0270
AC	0.0529	0.4535	0.5223	3.76	22.33	0.0021
BC	0.0006	0.0054	0.9437	1.70	10.10	0.0155
A^2^	48.37	414.66	<0.0001	171.87	1019.61	<0.0001
B^2^	55.99	480.06	<0.0001	35.57	211.01	<0.0001
C^2^	17.99	154.19	<0.0001	48.07	285.20	<0.0001
Residual	0.1166			0.1686		
Lack of fit	0.2065	4.19	0.1000	0.3162	5.46	0.0673
Pure error	0.0493			0.0579		

A is hydrolysis temperature, B is hydrolysis time, and C is hydrolysis pH. Response 1: *R*^2^ = 0.9959, Adj *R*^2^ = 0.9907, Pred *R*^2^ = 0.9489, Adeq Precision = 33.6087. Response 2: *R*^2^ = 0.9977, Adj *R*^2^ = 0.9947, Pred *R*^2^ = 0.9695, Adeq Precision = 50.0111.

**Table 3:** The energy and the amino acid residues involved in the interactions between peptides and myeloperoxidase (MPO) from the molecular docking study.

Peptide no.	Peptide Sequence	Consensus Score	−CDOCKER Interaction Energy	Interactions with A Chain of MPO	Interactions with B Chain of MPO	Interactions with C Chain of MPO	Interactions with D Chain of MPO
hLF1-11	GRRRRSVQWCA	-	120.282	ARG31, LEU33, PRO34	VAL30, ARG31, TRP32	THR159, ASN162, ARG323, LYS505	ALA152, CYS153, ILE160, ASN162, PHE439, LYS505
39	DTETGVPT	3	121.516	AGR31, TRP32	ALA28, PHE29. VAL30, ARG31, TRP32	ILE160, ARG323, LYS505	ALA152, CYS153, THR159, ILE160, ARG323, LYS505
1	NDNIQR	2	96.9148	ARG31, PRO34	ARG31, TRP32, ALA35	ILE160, LYS505	ILE160, ASN162, ARG323, LYS505
2	IELLLL	2	112.572	ARG31, TRP32, PRO34	ALA28, ARG31, PRO34, ALA35	ILE160, ASN162, PHE439	ALA152, CYS153, THR159, ILE160, ASN162, ARG323
4	LADEIR	2	101.61	ARG31, TRP32, PRO34	VAL30, ARG31, TRP32	ILE160, ASN162, LYS505	ALA152, CYS153, ARG323
5	LLKDQL	2	106.002	ALA28, VAL30, ARG31, PRO34	ALA28, TRP32	ILE160, ARG323, LYS505	ARG323, LYS505
6	LEILNT	2	95.5421	VAL30, ARG31	ALA28, ARG31	ALA152, CYS153, ILE160, ASN162, ARG323, LYS505	THR159, ILE160, ASN162, ARG323
8	QDPLNR	2	105.976	PHE29, ARG31, TRP32, PRO34, ALA35	TRP32	ILE160, ASN162	ASP321, ARG323, LYS505
9	QVQNVR	2	94.6778	ARG31, PRO34	ARG31, TRP32, PRO34	ILE160, ASN162, ARG323	ILE160, ASN162, SER319, ARG323, LYS505
10	GTELFR	2	97.3112	ARG31, TRP32, PRO34	ARG31, TRP32, ALA35	ASN162, ARG323, LYS505	ILE160, ASN162, ARG323, LYS505
12	ISAAELR	2	99.0441	ARG31, TRP32, ALA35	ARG31	ILE160, ARG323, LYS505	ILE160, ASN162, ARG323
13	FPSIVGR	2	89.5673	VAL30, ARG31	ALA28, PHE29, VAL30, ARG31, PRO34, ALA35	ILE160, ASN162, SER319, ARG323, LYS505	ALA152, CYS153, ILE160
14	LTGMAFR	2	90.0454	ARG31, LEU33	ARG31, TRP32, PRO34	ILE160, ARG323	ILE160, ASN162, ARG323, LYS505
16	FAPQLLT	2	105.345	ALA28, ARG31	ALA28, PHE29, VAL30, ARG31, PRO34	ILE160, ARG323	ILE160, ASN162, ARG323
20	LEVNLMT	2	106.656	PHE29, ARG31, TRP32, PRO34	ALA28, ARG31, TRP32, LEU33	ILE160, ASN162, ARG323, LYS505	ALA152, ILE160, ASN162, ARG323
22	HLQLAIR	2	98.3054	ALA28, ARG31, TRP32	ARG31, TRP32	THR159, ILE160, ARG323	SER156, THR159, ILE160, ASP321, ARG323
23	IAKEGFA	2	92.2588	PHE29, ARG31, LEU33, PRO34	ARG31, TRP32	THR159, ARG323, LYS505	ILE160, ASN162
24	NLNPTTK	2	95.8118	ARG31, TRP32, PRO34	PHE29, ARG31, ALA35	CYS153, ARG323	ILE160, ASN162, ARG323
25	VSHYSTK	2	90.9066	ARG31	ALA28, ARG31, TRP32, ALA35	ILE160, ASN162, ARG323	ALA152, IEL160, ARG323
28	GLLLHWS	2	99.7525	ALA28, VAL30, ARG31, TRP32	ALA28, PHE29, ARG31, TRP32, PRO34	ALA152, CYS153, ILE160, ARG323, LYS505	ILE160, ASN162, ARG323, LYS505
29	LQLISAG	2	89.9116	ALA28, ARG31, TRP32	VAL30, ARG31	CYS153, ILE160, ARG161, ASN162, ASP321, ARG323, LYS505	ILE160, ASN162
30	QCELNFK	2	103.547	ARG31, PRO34	-	ILE160, ASN162, ARG323, LYS505	ALA152, CYS153, ASN162, ARG323
31	GIHETTY	2	120.866	VAL30, ARG31, PRO34	ALA28, ARG31, TRP32, PRO34	THR159, ILE160, ASN162, ARG323	ALA152, ILE160, ARG323
45	VASFSTHK	2	97.4559	ALA28, ARG31	ALA28, VAL30, ARG31, TRP32	ILE160	ALA152, SER319, ARG323, LYS505
46	AVAGLGIL	2	94.3888	ARG31, TRP32	PHE29, ARG31, TRP32	ALA152, CYS153, ILE160, ASN162, ARG323, LYS505	CYS153, ILE160, ASN162, ARG323
48	VGDEAQSK	2	114.397	ARG31	ALA28, TRP32	YHR159, ILE160, ARG323	THR159, ILE160, ASN162, ARG323, LYS505
49	SSSVVAAL	2	95.2041	ARG31	ARG31, TRP32	ASN162, ARG323, LYS505	ALA152, CYS153, ASN162, ARG323
50	FAGDDAPR	2	113.395	ARG31, TRP32, PRO34	PHE29, VAL30, ARG31, TRP32, PRO34	ILE160, ASN162, ARG323	THR159, ILE160, ASN162, ARG323
55	KTKDDMHL	2	111.666	ARG31, TRP32, PRO34, ALA35	ARG31, TRP32, PRO34	ILE160, ARG323, LYS505	CYS153, THR159, ILE160, ARG323
58	VIPELNGK	2	108.917	ALA28, ARG31	ALA28, ARG31, ALA35	ARG323, LYS505	ILE160, ASN163, ARG323
62	GFAGDDAPR	2	128.195	ALA28, ARG31, TRP32, ALA35	ALA28, ARG31, TRP32	ILE160, SER319, ARG323, LYS505	ILE160, ASN162, ARG323
64	VATVGPISV	2	96.6785	ALA28, ARG31, PRO34	ALA28, VAL30, ARG31, TRP32, PRO34	THR159, ILE160, ARG323	ILE160, ASN162, ARG323, LYS505
68	SSSVALHKH	2	78.6093	ARG31, TRP32, PRO34	ARG31, TRP32	PRO151, CYS153, THR159, ILE160, ARG161, ASN162, ARG323, LYS505	THR159, ILE160, ARG323
71	GGKNLDELE	2	145.154	ARG31, PRO34	ALA28, PHE29, ARG31, TRP32	ILE160, ARG323	CYS153, SER156, THR159, ILE160, ASN162, ASP321, ARG323, LYS505
111	VGSSFVGGFG	2	103.741	VAL30, ARG31, TRP32	ALA28, ARG31	ILE160, ASN162, LYS505	PRO151, CYS153, SER156, THR159, ILE160, ARG161, ASN162, ARG323
112	DVNSLKSALA	2	103.755	ARG31, TRP32	VAL30, ARG31, TRP32	CYS153, ILE160, ARG323, LYS505	ALA152, CYS153, ILE160, ARG161, ASN162
118	IGGIGTVPVGR	2	106.314	ARG31, PRO34, SER42	VAL30, ARG31, TRP32, PRO34	PRO151, ALA152, CYS153, SER156, ILE160, ASN162, ASN317, SER319	ARG323, ARG438, PHE439
125	AGLFVSSFFSV	2	116.238	ALA28, ARG31, TRP32	ALA28, VAL30, ARG31, PHE41	CYS153, LYS308, ASP321, ARG323, PHE439, LYS505	CYS153, SER156, THR159, ILE160, ARG323
148	LTASGPSIGARP	2	122.053	ARG31	ALA28, TRP32, ASP39	ARG323, LYS505, PHE439	PRO123, ALA152, CYS153, GLY155, SER156, THR159, ILE160, ARG161, ASN162
182	ILVGAAVCFFCLILA	2	116.4	ALA28, PRO103	ARG31, TRP32	PRO151, CYS153, PRO154, GLY155, SER156, THR159, ILE160, ARG161, ASN162, ARG323	ILE160, ASN162, LYS308, ASP321, ARG323, PHE439, LYS505
183	STAGDTHLGGEDFDNR	2	140.863	ALA28, TRP32	ARG31, PRG41, SER42, LEU43	LYS308, ASP321, ARG323, PHE439, CYS440, CYS497, CYS505, LYS505	PRO151, CYS153, GLY155, THR159, ILE160, ARG161, ASN162, ARG323
7	NWDLVG	1	99.433	PHE29, VAL30, ARG31	ALA28, PHE29, VAL30, ARG31, TRP32	ARG323	ARG323
11	LLVKLL	1	86.1708	ALA28, VAL30, ARG31, TRP32, PRO34	PHE29, VAL30, ARG31, PRO34	ALA152, CYS153, ASN162, ARG323, LYS505	ILE160, ASN162
15	FPGIADR	1	88.1065	VAL30, ARG31	PHE29, VAL30, ARG31, TRP32,	ASN162, ARG323	ILE160, SER319, ARG323
17	IIAPPER	1	96.3472	VAL30, ARG31, TRP32, PRO34	ALA28, ARG31, TRP32	ALA152, CYS153, IEL160, ASN162, ARG323, LYS505	ILE160, ARG323, LYS505
21	LIILELL	1	98.6814	ALA28, ARG31, TRP32, LEU33, PRO34	ALA28, VAL30, ARG31, PRO34	ILE160, ARG323, LYS505	ALA152, CYS153, ILE160, ASN162
26	NLLNIPK	1	86.0812	VAL30, ARG31, PRO34	ARG31, TRP32, PRO34	ILE160, ARG323	THR159, ILE160
27	SFRENNT	1	102.146	ARG31, PRO34	PHE29, ARG31, TRP32	ILE160, ASN162, LYS505	ASN162, ASP321, ARG323, LYS505
32	TEAPLNPK	1	101.636	VAL30, ARG31, TRP32, PRO34	ALA28, ARG31, PRO34	THR159, ILE160, ASN162, LYS505	ILE160, ASN162, ARG323
34	IILLLLV	1	89.3092	ALA28, VAL30, ARG31, TRP32, PRO34, ALA35	VAL30, TRP32, PRO34	ILE160, ASN162, ARG323, LYS505	ILE160
35	SFTTTAER	1	111.511	ARG31, TRP32	PHE29, VAL30, ARG31, TRP32	THR159, LYS505	CYS153, SER156, THR159, ASN162
37	TLEEEKLQ	1	127.638	ARG31, TRP32, PRO34	ALA28, TRP32, LEU33, PRO34	ILE160, ASN162, ARG323, LYS505	ALA152, CYS153, THR159, ILE160, ARG323
40	FLNNNALT	1	88.3879	ARG31, TRP32	ARG31	THR159, ARG323	ILE160, ASN162, SER319, VAL320, ARG323
41	LEVLGVPA	1	96.8835	PHE29, VAL30, ARG31, TRP32, LEU33, PRO34	ALA28, VAL30, ARG31, PRO34	CYS153, THR159, ILE160, ASN162, ARG323, LYS505	ILE160
42	KLDEKIVQ	1	102.044	VAL30, ARG31, LEU33, PRO34	ALA28, ARG31, PRO34, SER42	ARG323	ALA152, CYS153, ILE160, ARG161, ASN162, SER319, ARG323, LYS505
44	TVPIYEGY	1	88.1488	VAL30, TRP32, SER42	ALA28, ARG31, TRP32	ALA152, CYS153, SER156, THR159, ILE160, ARG161, ASN162	ASN162
52	SVKNTAGL	1	89.225	ARG31, LEU33	ALA28, ARG31	CYS153, ASN162, ARG323	ILE160, ARG323, LYS505
53	ADINAADQ	1	121.529	ARG31	ARG31, TRP32, LEU33, PRO34	ALA152, CYS153, ILE160, ASN162	THR159, ILE160, ASN162, ARG323, LYS505
54	KTWVKELQ	1	80.8878	ALA28, ARG31, PRO34, SER42	ARG31, TRP32,	CYS153, THR159, ILE160, ASN162	ILE160, ARG323, LYS505
57	EEQVAAIR	1	124.143	ARG31, TRP32	PHE29, ARG31, PRO34, ALA35	THR159, ILE160, ARG323, PHE439	ALA152, CYS153, THR159, ILE160, SER319, ARG323, LYS505
63	MGSTLIMLL	1	120.627	VAL30, ARG31, TRP32, LEU33, PRO34	ALA28, VAL30, ARG31	ILE160, ASN162, PHE439, LYS505	PRO151, CYS153, SER156, THR159, ILE160, ANS162
70	LGKTVPDDV	1	88.3103	ARG31, TRP32, LEU33, PRO34	PHE29, ARG31, PRO34	ILE160, ASN162, ARG323, LYS505	PRO151, ALA152, CYS153, ILE160, ARG161, ANS162, ARG323
73	QVITIGNER	1	113.155	ALA28, VAL30, ARG31, PRO34	ALA28, VAL30, ARG31, LEU33, ALA35	ARG323, LYS505	ALA152, PRO154, GLY155, SER156, THR159, ILE160, ANS162, SER319, ARG323, LYS505
74	LSDLSPFPG	1	82.875	TRP32	ALA28, PHE29, ARG31, TRP32, PRO34	ALA152, CYS153, THR159, ILE160, ASN162, ARG323, LYS505	ALA152, CYS153, THR159, ILE160, ARG161, ANS162, PHE439
78	VGYDALTDQ	1	133.602	ALA28, VAL30, ARG31, TRP32	ARG31, PRO34, ALA35	ALA152, CYS153, ARG323, LYS505	PRO151, CYS153, SER156, THR159, ILE160, ARG161, ANS162, ARG323, LYS505
81	IEAIDQVGS	1	124.082	ARG31, LEU33	VAL30, ARG31, SER42	CYS153, THR159, ASN162, ARG323, PHE439	CYS153, GLY155, SER156, THR159, ILE160, ASN162, ARG323, LYS505
82	MKNPKASVL	1	48.301	ARG31, TRP32	-	ILE160, LYS505	SER156, THR159, ILE160, ASN162, ARG323, PHE439
84	LIIIIAAMT	1	80.6012	VAL30, ARG31, TRP32	ALA28, ARG31, PRO34, ALA35	ILE160, ASN162	CYS153, ILE160, ASN162
90	AGFAGDDAPR	1	118.225	ARG31, PRO34	ALA28, VAL30, ARG31, PRO34	ARG323	CYS153, SER156, THR159, ILE160, ASN162, ARG323
91	DAVTYTEHAK	1	130.855	VAL30, ARG31, PRO34	ARG31, PHE41	ILE160, ARG323, LYS505	CYS153, PRO154, GLY155, SER156, THR159, ILE160, ARG161, ASN162, ARG323, PHE439
94	VLMILPSVTG	1	80.7513	VAL30, ARG31, PRO34, ALA35	ALA28, ARG31, TRP32, LEU33, PRO34	ILE160, ASN162, ARG323	PRO151, ALA152, CYS153, GLY155, THR159, ILE160, ARG161, ARG323, PHE439
107	DSGLLTPESV	1	88.9027	ALA28, ARG31, SER42	ARG31, TRP32, PRO34	CYS153, GLY155, SER156, THR159, ILE160, ARG161, ASN162, ARG323, LYS505	ILE160
109	VEQEILETGI	1	95.3997	ARG31, TRP32	VAL30, ARG31, TRP32, PHE41	THR159, ASN162, ARG323, LYS505	ALA152, PRO154, GLY155, SER156, THR159, ILE160, ARG161, ANS162, ARG323, LYS505
113	SDDLDLGQVG	1	63.6376	ARG31, PRO34	VAL30, ARG31, TRP32, PRO34	ARG323, LYS505	ARG148, CYS153, GLY155, SER156, THR159, ILE160, ARG161, ANS162
114	HQGVMVGMGQK	1	108.89	ALA28, VAL30, ARG31, TPR32	ARG31, ASP39, SER42	THR159, PHE439, LYS505	PRO151, ALA152, CYS153, THR159, ILE160, ARG161, ANS162
122	LSAAGLEAGNV	1	104.596	ARG31, TRP32	ARG31, SER42	THR159, ARG323	ALA152, CYS153, PRO154, GLY155, SER156, THR159, ILE160, ANS162, SER319, ASP321, ARG323
123	ATAASSSSLEK	1	107.999	ARG31, TRP32, PRO34	VAL30, ARG31, ASP39, SER42	ARG323, PHE439, LYS505	CYS153, GLY155, THR159, ILE160, ARG161, ASN162
124	VVVYGGVAVNH	1	58.4842	ARG31	ALA28, ARG31, PRO34, PHE41, SER42	ASN162, ARG323, PHE439	ARG148, PRO151, ALA152, CYS153, SER156, THR159, ILE160, ANS162, ARG323
130	KAGSERNVLIF	1	127.874	ARG31, TRP32, PRO34, ALA35, ASP39, SER42	ARG31, PRO34	CYS153, PRO154, GLY155, SER156, SER156, THR159, ILE160, ARG161, ASN162, PHE439, LYS505	ILE160, ARG161, ARG323, LYS505
137	IGLFGGAGVGK	1	104.383	ALA28, ARG31, PRO34	PHE29, ARG31, PRO34, PHE41	ARG323, PHE439, LYS505	PRO151, CYS153, SER156, THR159, ILE160, ARG323
139	TLSIQNDQASQR	1	116.278	ARG31, TRP32	-	ILE160, ARG161, ASN162, ARG323, PHE439, CYS497, LYS505	ARG148, CYS153, ARG161, ASN162, SER156, THR159, ILE160, ARG323
142	DSYVGDEAQSKR	1	91.8078	PRO34	ARG31	ARG323, PHE439	CYS153, GLY155, SER156, THR159, ILE160, ARG161, SER319, ARG323, ARG504
152	YISHIELAFSSV	1	122.83	ARG31, TRP32, PRO34	ALA28, TRP32	ARG323, PHE439, LYS505	PRO151, CYS153, PRO154, GLY155, SER156, THR159, ILE160, ASN162
154	EGGGIVESIGEG	1	150.197	ARG31, TRP32, PRO34	ARG31, TRP32, SER42	ALA152, ARG323, LYS505	ALA152, CYS153, SER156, ILE160, ARG161, ARG323, PHE439
155	CRPGALESGPAL	1	91.9951	ALA28, PRO34	ARG31	ALA152, CYS153, PRO154, GLY155, SER156, ILE160, ARG161, ASN162, ARG323	ILE160, ARG323, PHE439, LYS505
156	FNLWGLSCSSLL	1	88.0987	VAL30, ARG31, TRP32, PRO34	VAL30, ARG31, PRO34, SER42, LEU43	ASN162	PRO123, ARG148, PRO151, CYS153, GLY155, SER156, THR159, ILE160, ARG161, ASN162, ASP321, ARG323
161	AKCGAYQGQVLIF	1	114.346	ARG31, TRP32	TRP32	ILE160, ASN162, LYS308, ARG323, PHE439	CYS153, SER156, THR159, ILE160, ARG161, ASN162
169	FTQAGSEVSALLGR	1	130.617	ARG31, PRO34	VAL30, ARG31	-	CYS153, GLY155, SER156, ILE160, ARG161, ASN162, ARG323, PHE439
170	TTGIVLDSGDGVTH	1	95.5298	ARG31, PRO34	VAL30, ARG31, PRO34, SER42	ASN162, ARG323, ARG438, PHE439, GLY441	PRO123, PRO151, CYS153, PRO154, GLY155, SER156, THR159, ILE160, ARG161, SER319, ARG323
172	AHGGYSVFAGVGER	1	106.868	VAL30, ARG31	PHE29, VAL30, ARG31, TRP32, SER42	CYS153, ASN162, PHE439, LYS505	ARG148, ALA152, CYS153, PRO154, GLY155, THR159, ARG161, ASN162, ARG323, LYS505
177	ELPDGQVITIGNER	1	113.61	ARG31, TRP32, PRO34, ASP39, SER42	ALA28, ARG31, TRP32, LEU33, PRO34	CYS153, GLY155, SER156, THR159, ILE160, ARG161, ASN162, SER319. ARG323, LYS505	THR159, ASP321, ARG323
178	SAYLVLTITIAAMT	1	111.103	ARG31, TRP32, PRO34	ALA28, ARG31, PHE41	CYS153, ILE160, ARG438, PHE439, LYS505	ALA152, CYS153, GLY155, SER156, THR159, ILE160, ARG161, ASN162, ARG323
180	WEDLANEIQEELNK	1	107.719	ARG31, TRP32	VAL30, ARG31, TRP32, PRO34, PHE41	THR159, ARG438, CYS440, GLY441, LYS505	PRO123, ARG148, PRO151, ALA152, CYS 153, THR159, ILE160, ARG161, ASN162, ARG323, LYS505
186	EQEGGLGNFMNFMKENG	1	163.68	TRP32, PRO34	ALA28, PHE29, ARG31, LEU33, PRO34, PHE41, PRO101, PRO103	ILE160, ARG323, PHE439, CYS440	PRO124, PRO151, CYS153, SER156, THR159, ILE160, ARG161, ASN162, SER319
51	LILLIRAK	0	74.004	ALA28, PHE29, ARG31, TRP32, PRO34	ALA28, ARG31, PRO34, SER42	ILE160, PHE439, LYS505	ALA152, CYS153, SER156, ILE160, ARG161, ANS162
59	LARDKAAN	0	94.6022	ARG31, TRP32	PHE29, VAL30, ARG31, LEU33	THR159, ILE160, ASN162, ARG323, LYS505	THR159
67	VLFPLTTLQ	0	72.9901	VAL30	ARG31, TRP32, SER42	CYS153, LYS505	PRO151, ALA152, CYS153, SER156, ARG161, ANS162, ARG323
72	SMSFFGLIM	0	86.9571	-	ALA28, ARG31, TRP32, PRO34	ASN162, ARG323, PHE439	PRO151, ALA152, ILE160, ARG161, ASN162, ASP321, LYS505
85	ISGLIYEETR	0	75.3523	ARG31, TRP32, SER42	ALA28, ARG31, LEU33, PRO34	CYS153, PRO154, GLY155, SER156, THR159, ILE160, ASN162, ARG323, LYS505	CYS153, ARG323
99	VVTSGNSLNG	0	93.2669	VAL30, ARG31, TRP32	PHE29, ARG31, TRP32	ILE160, ARG323, PHE439	ALA152, CYS153, ILE160, ANS162, LYS505
117	VEIIANDQGNR	0	114.499	ARG31, PRO34	VAL30, ARG31, SER42	ASN162, ARG323, PHE439	ARG148, CYS153, GLY155, SER156, THR159, ILE160, ARG161, ASN162, ARG323
121	PTKVGINGFGR	0	81.1789	ALA28, PHE29, ARG31, TRP32	ALA28, SER42	LYS505	CYS153, GLY155, SER156, THR159, ILE160
140	ELISNASDALDK	0	74.9956	ARG31, TRP32, TRP47	LEU33	ARG148, PRO151, CYS153, PRO154, THR159, ILE160, ARG161, ASN162, ARG323, LYS505	ARG323
162	LLTLATCVGDGPA	0	72.2681	VAL30, ARG31, TRP32, SER42	ARG31	CYS153, GLY155, SER156, THR159, ILE160, ARG161, ASN162	CYS153, LYS505
164	RSLGDFACQLEHL	0	75.728	ARG31, SER42	TRP32	PRO151, CYS153, GLY155, SER156, THR159, ILE160, ARG161, LYS505	THR159, LYS308, ARG323, PHE439, CYS440
171	LISWYDNEYGYSNR	0	137.019	ALA28, ARG31, TRP32, PRO34	PHE29, PHE41, SER42	THR159, ASN162	ALA152, CYS153, PRO154, SER156, THR159, ARG161, ASN162, VAL320, ASP321, ARG323
191	LVQDVANNTNEEAGDGTTTATVLAR	0	120.381	VAL30, ARG31, TRP32, LEU33	VAL30, TRP32, LEU43	THR159, ILE160, PHE439	CYS153, GLY155, SER156, ASN157, THR159, ILE160, ARG161, ASN317, SER319, ARG323, ARG504, LYS505
192	AQTISYEVSMALVLLFPLFLGGSFSF	0	185.828	TRP32	ARG31, TRP32, PRO34, SER42	ASN162, ARG323, PHE439, GLY441, PRO443, LYS505	GLN122, PRO123, CYS153, PRO154, GLY155, SER156, THR159, ILE160, ARG161, ASN162, ARG323, LYS505

## Supplementary Materials

The following are available online at http://www.mdpi.com/1660-3397/17/2/116/s1, Table S1: The peptide sequences characterized in hydrolysate fraction of MW < 3kDa.
