# Supplementary material for: Antioxidative Peptides from Proteolytic Hydrolysates of False Abalone (Volutharpa ampullacea perryi): Characterization, Identification, and Molecular Docking
Source: Mar Drugs. 2019 Feb 13;17(2):116. doi: 10.3390/md17020116 (PMC6409897; doi:10.3390/md17020116)
Supplement: Supplementary file 1 [file marinedrugs-17-00116-s001.pdf]

Supplement Table 1

The peptide sequences characterized in hydrolysate fraction of MW < 3kDa.

Peptide No.	Peptide sequence
1	NDNIQR
2	IELLLL
3	LLNTLK
4	LADEIR
5	LLKDQL
6	*LEILNT*
7	NWDLVG
8	QDPLNR
9	QVQNVR
10	GTELFR
11	LLVKLL
12	ISAAELR
13	FPSIVGR
14	LTGMAFR
15	FPGIADR
16	FAPQLLT
17	IIAPPER
18	FPLLLFR
19	KIDNYTK
20	LEVNLMT
21	LIILELL
22	HLQLAIR
23	IAKEGFA
24	NLNPTTK
25	VSHYSTK
26	NLLNIPK
27	SFRENNT
28	GLLLHWS
29	LQLISAG
30	QCELNFK
31	GIHETTY
32	TEAPLNPK
33	IIIIILII
34	IILLLLV
35	SFTTTAER
36	VFLENVIR
37	TLEEEKLQ
38	LLIILELL
39	DTETGVPT
40	FLNNNALT
41	LEVLGVPA
42	KLDEKIVQ

43	LIMMLGWI
44	TVPIYEGY
45	VASFSTHK
46	AVAGLGIL
47	ESILQRIW
48	VGDEAQSK
49	SSSVVAAL
50	FAGDDAPR
51	LILLIRAK
52	SVKNTAGL
53	ADINAADQ
54	KTWVKELQ
55	KTKDDMHL
56	DLTQLPYT
57	EEQVAAIR
58	VIPELNGK
59	LARDKAAN
60	LLIPSLLLL
61	TLSDYNIQK
62	GFAGDDAPR
63	MGSTLIMLL
64	VATVGPISV
65	AGLQFPVGR
66	LLIILLLV
67	VLFPLTTLQ
68	SSSVALHKH
69	SDVYMVTSL
70	LGKTVPDDV
71	GGKNLDELE
72	SMSFFGLIM
73	QVITIGNER
74	LSDLSPFPG
75	LLLLFAVGN
76	LTESQIKIW
77	LEDELLAEK
78	VGYDALTDQ
79	KPKESKDPF
80	VTFQQSAER
81	IEAIDQVGS
82	MKNPKASVL
83	RSITRRVDE
84	LIIIIAAMT
85	ISGLIYEETR
86	DESGPSIVHR
87	YSQLENEFDR

88	IQLLEEDLER
89	LAITEVDLER
90	AGFAGDDAPR
91	DAVTYTEHAK
92	LTEEQIAEFK
93	LLIPSLLLLL
94	VLMILPSVTG
95	ELAFQSDEDR
96	LVGVFSDVQW
97	GWGQWIIQPK
98	LSNLLSKRVS
99	VVTSGNSLNG
100	AVFPSIVGRP
101	STTTGHLIYK
102	KDQVVYSVAA
103	LIATVALGIY
104	LLTEAPLNPK
105	VAPEEHPVLL
106	GYSFTTTAER
107	DSGLLTPESV
108	QTEKVVFCRD
109	VEQEILETGI
110	IASAPSVNVR
111	VGSSFVGGFG
112	DVNSLKSALA
113	SDDLDLGQVG
114	HQGVMVGMGQK
115	IWHHTFYNELR
116	ILLIIIIILII
117	VEIIANDQGNR
118	IGGIGTVPVGR
119	EITALAPSTMK
120	DSYVGDEAQSK
121	PTKVGINGFGR
122	LSAAGLEAGNV
123	ATAASSSSLEK
124	VVVYGGVAVNH
125	AGLFVSSFFSV
126	LLLLIASAHSV
127	LFKDYLLDSGL
128	DAQPKTKYDVP
129	AVFPSIVGRPR
130	KAGSERNVLIF
131	LTLSGGQKQRI
132	ELDSLAVPLQQ

133	DRASALIAQAI
134	ALEDADTKNDP
135	MIGINVFNLLG
136	LQACIDLIEKP
137	IGLFGGAGVGK
138	LLEKLATEFGL
139	TLSIQNDQASQR
140	ELISNASDALDK
141	EHALLAYTLGVK
142	DSYVGDEAQSKR
143	YYVTIIDAPGHR
144	PWIAAQDSSTVE
145	EVDEQMLNVQNK
146	GEASLILITLGA
147	KGENELCDIITQ
148	LTASGPSIGARP
149	MSIVLVAFEGAP
150	LLVSPAATMPVD
151	WSGLVETALNLL
152	YISHIELAFSSV
153	IAENELCDIITQ
154	EGGGIVESIGEG
155	CRPGALESGPAL
156	FNLWGLSCSSLL
157	GTIDLQMPKVLQ
158	KDADGALLTMLI
159	QEYDESGPSIVHR
160	TVLIMELINNVAK
161	AKCGAYQGQVLIF
162	LLTLATCVGDGPA
163	SAAGVVVLPKTED
164	RSLGDFACQLEHL
165	LGVYFTFLQGCEY
166	QNVHLMAGMTQPK
167	KPGGDMIIPDPKQ
168	FKKAAAGIDDIP
169	FTQAGSEVSALLGR
170	TTGIVLDSGDGVTH
171	LISWYDNEYGYSNR
172	AHGGYSVFAGVGER
173	NSSYFVEWIPNNVK
174	RSVFLSKGMLMLLP
175	GVVDSEDLPLNISR
176	AREYDRILSNRMNR
177	ELPDGQVITIGNER

178	SAYLVLTITIAAMT
179	MQIHAHGHLVYGMG
180	LLASMPLLVSLAWC
181	WEDLANEIQEELNK
182	ILKVLESKRTCCR
183	ILVGAAVCFFCLILA
184	STAGDTHLGGEDFDNR
185	MSATFIGNSTAIQELFK
186	TEKGILSSIMGALGGIV
187	EQEGGLGNFMNFMKENG
188	DVCQPNLLHPLCQECEG
189	VAPEEHPVLLTEAPLNPK
190	IPSAVGYQPTLATDMGTMQER
191	QTQTFTTYSDNQPGVLIQVYEGER
192	LVQDVANNTNEEAGDGTTTATVLAR
193	AQTISYEVSMALVLLFPLFLGGSFSF
